# Pseudoalteromonas holothuriae sp. nov., isolated from the sea cucumber Holothuria forskali

**DOI:** 10.1099/ijsem.0.006601

**Published:** 2025-02-24

**Authors:** Marion Yvin, Estelle Mühle, Olivier Chesneau, Hélène Laguerre, Benjamin Brillet, Yannick Fleury, Camille Jégou, Peter Kämpfer, André Lipski, Alexis Criscuolo, Dominique Clermont, Patrick Le Chevalier

**Affiliations:** 1 Université de Brest, Laboratoire de Biotechnologie et Chimie Marines, LBCM, EMR-CNRS 6076, F-29000 Quimper, France; 2 Université Bretagne Sud, IRDL, UMR CNRS 6027, F-56100 Lorient, France; 3 Institut Pasteur, Université Paris Cité, Collection de l’Institut Pasteur-CIP, F-75015 Paris, France; 4 Institut für Angewandte Mikrobiologie, Justus-Liebig, Universität Giessen, Heinrich-Buff-Ring 26 (IFZ), 35392 Giessen, Germany; 5 Lebensmittelmikrobiologie und -hygiene, Institut für Ernährungs- und Lebensmittelwissenschaften, Rheinische Friedrich-Wilhelms-Universität Bonn, Friedrich-Hirzebruch-Allee 7, 53115 Bonn, Germany; 6 Institut Pasteur, Université Paris Cité, GIPhy – Genome Informatics and Phylogenetics, Biological Resource Center of Institut Pasteur, F-75015 Paris, France

**Keywords:** coelomic fluid, *Pseudoalteromonas*, seawater, sea cucumber

## Abstract

Two motile bacterial strains, designated as cfHf56-1^T^ and SW 252, were isolated from the coelomic fluid of *Holothuria forskali* and from the surrounding seawater at the animal sampling site, respectively. The sea cucumber was collected in the Glénan archipelago (Brittany, France). Strains cfHf56-1^T^ and SW 252 were Gram-stain-negative, non-spore-forming and rod-shaped bacteria. Colonies made on marine agar plates were brown in color. The pH and temperature ranges for growth were 7–8 and 18–30 °C, respectively, in marine broth. The major fatty acids were 16 : 1 cis9 and 16 : 0. Phylogenetic analyses evidenced that both strains belong to a novel species in the genus *Pseudoalteromonas*. The strains were closely related to the type strains of *Pseudoalteromonas caenipelagi*, *Pseudoalteromonas byunsanensis* and *Pseudoalteromonas amylolytica,* with 75–78 % ANI and 19–21 % dDDH values. In this context, cfHf56-1^T^ (= CIP 111854^T^ = CECT 30642^T^) is considered as the type strain of the novel species for which the name *Pseudoalteromonas holothuriae* sp. nov. is proposed. The genome of the type strain is characterized by a size of 5.1 Mbp and a G+C content of 40.5%.

## Introduction

The genus *Pseudoalteromonas* was first proposed by Gauthier *et al*. in 1995, causing the division of *Alteromonas* into two genera. Most of the previously identified *Alteromonas* strains were consequently, at this time, reclassified as *Pseudoalteromonas* based on the analysis of 16S rRNA gene sequences [1]. Further phylogenetic studies using 16S rRNA signature sequences erected the genus *Pseudoalteromonas* in 2004 as the type genus of the *Pseudoalteromonadaceae* family, *Alteromonadales* order, *Gammaproteobacteria* class [2]. To date, there are 49 validly recognized *Pseudoalteromonas* species that were listed by ICSP (https://lpsn.dsmz.de/genus/pseudoalteromonas). Phenotypically, these bacteria are aerobic and heterotrophic Gram-stain-negative rods, motile and sometimes pigmented. They are strictly related to marine environments, isolated from various habitats such as sediments [3] or seawater [4]. They are also frequently found in association with marine macro-organisms like tunicates [5], bivalves [6], corals [7], sponges [8] and algae [9]. The complex association between animal or plant host and its associated microbiota has been theorized by Rosenberg as a holobiont [10]. *Pseudoalteromonas* strains are known to produce a wide variety of bioactive compounds and pigments, some of which could contribute to host fitness [11].

Marine invertebrates such as sea cucumbers (Echinodermata) are important models to understand the association between micro-organisms and their hosts [12]. The presence of gut and coelomic microbiota was verified in sea cucumbers, but little is known about the role of micro-organisms [13]. In the coelomic fluid, bacteria exhibiting antibacterial and antifungal activities were isolated [13,14]. The coelomic fluid is enclosed in the coelomic cavity and is composed of immune cells (coelomocytes). This internal circulatory system mediates the immune response and is not directly connected to the surrounding seawater [15]. Thus, several questions are raised about the selection of the microbiota by the host or its role in the animal’s vital functions.

Through our investigation of the microbial communities associated with the sea cucumber *Holothuria forskali*, two bacterial strains were recently isolated. Here, we present a polyphasic approach, assigning the taxonomic position of these two strains: cfHf56-1^T^ (= CIP 111854^T^ = CECT 30642^T^) from the coelomic fluid of the sea cucumber and SW 252 (= CIP 111951) from its surrounding environment.

## Sampling and isolation

Wild sea cucumbers (*n*=15) were collected from rocky substrates at a depth of around 15 m, during June 2019 in the Glénan archipelago (47°43′57.76″N, 04°00′50.99″W, Brittany, France), located in the Natura 2000 zone (FR5300023). Ambient seawater samples (15 ml) were collected at the same time. Coelomic fluid samples were aseptically collected from the coelomic cavity using a 25-gauge needle attached to a 10 ml syringe.

To isolate cultivable bacteria, 100 µl of a tenfold dilution of coelomic fluid or ambient seawater was spread onto Marine Agar (Marine Broth® Difco 2216+1.5% Agar, Thermo Fisher Scientific, Waltham, USA) using sterilized solid-glass beads (Thermo Fisher Scientific, Waltham, USA). After 72 h of incubation at 18 °C, the isolated bacteria were screened for their antibacterial activities against human and aquaculture pathogens. Agar-well diffusion assays demonstrated that both cfHf56-1^T^ (isolated from coelomic fluid) and SW 252 (isolated from ambient seawater) could inhibit the growth of *Staphylococcus aureus*, *Vibrio anguillarum*, *Vibrio harveyi* and *Vibrio pectinidae* [16]. Thus, these two screened isolates that looked very similar on MA were kept for further characterization. Long-term preservation of the strains was made by adding sterile glycerol to 1 ml marine broth (MB) pure culture (25 % v/v) in cryogenic vials before storage at −80 °C.

## 16S rRNA phylogeny

Genomic DNA was extracted and purified using the NucleoSpin Microbial DNA kit (Macherey-Nagel, Düren, Germany). The 16S rRNA gene was amplified by PCR using universal primers 27F (5′-GAGTTTGATCMTGGCTCAG-3′) and 1492R (5′-GNTACCTTGTTACGACTT-3′) [17], along with OneTaq DNA Polymerase (New England Biolabs, Ipswich, USA). PCR products were sequenced using the Sanger method (Eurofins Genomics, Germany). The 16S rRNA sequence of strain cfHf56-1^T^ was a continuous stretch of 1406 bp (accession number MN942006). This sequence, fully identical to that of SW 252, was submitted to blastn analyses against GenBank and EzBioCloud databases. It was observed to be closely related to that of *Pseudoalteromonas caenipelagi* JBTF-M23^T^ by sharing 98.3% nucleotide identity.

**Fig. 1.**
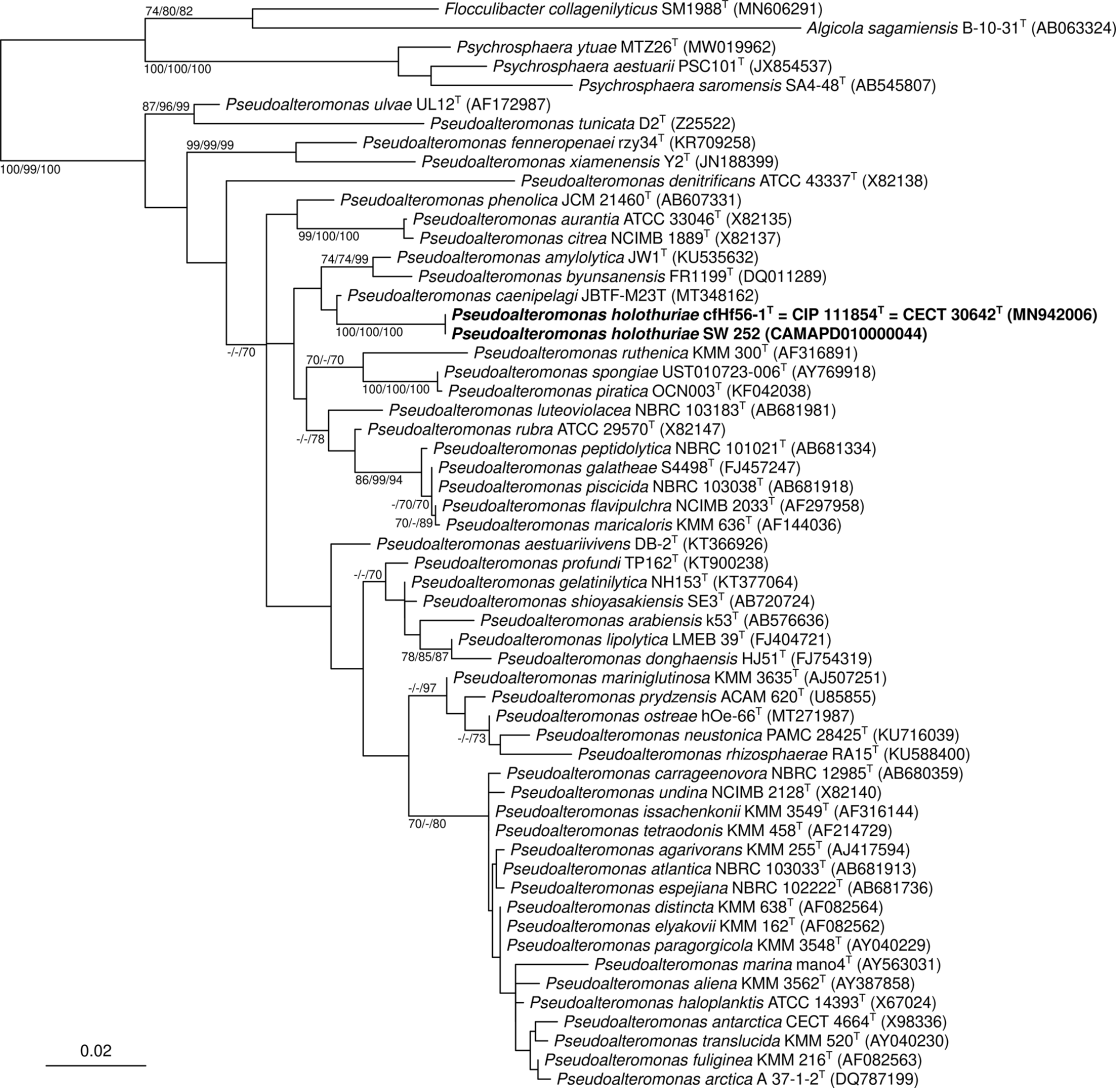
ML phylogenetic tree based on 16S rRNA sequences showing the phylogenetic positions of strains cfHf56-1^T^ and SW 252 among the type strains of genus *Pseudoalteromonas*. Type strains of *Algicola*, *Flocculibacter* and *Psychrosphaera* were used as an outgroup. Sequence accessions are indicated between parentheses. Branch supports (>70 %) derived from bootstrap analyses (500 replicates) using three phylogenetic inference criteria (ML/MP/ME) are indicated. Bar, 0.02 nucleotide substitutions per site.

A multiple sequence alignment of all 16S rRNA gene sequences of *Pseudoalteromonas* type strains was inferred using sina version 1.2.11 [18], along with five outgroup sequences of *Algicola*, *Flocculibacter* and *Psychrosphaera* type strains. The resulting multiple sequence alignment was manually corrected, leading to 1365 aligned characters. Phylogenetic reconstructions were carried out by optimizing maximum likelihood (ML), maximum parsimony (MP) and minimum evolution (ME) criteria. ML analysis was performed using IQ-TREE version 2.0.6 [19] with evolutionary model HKY+F+R4, derived by minimizing the Bayesian information criterion. MP inference was carried out using dnapars version 3.6. FastME version 2.1.6.2 [20] was used to infer an ME tree with the F81+G correction. For each ML, MP and ME criterion, bootstrap branch supports were estimated (500 replicates). Both strains cfHf56-1^T^ and SW 252 were clustered with the type strains of *P. caenipelagi*, *P. amylolytica* and *P. byunsanensis* within the genus *Pseudoalteromonas* (Fig. 1). According to these preliminary results, *P. caenipelagi* CIP 111909^T^ [21], *P. byunsanensis* CIP 109024^T^ [3] and *P. amylolytica* CIP 111901^T^ [22], hosted at the Collection de l’Institut Pasteur (CIP; Paris, France), were used as reference strains for genotypic and phenotypic characterizations. All strains were cultivated on MA.

## DNA extraction and genome features

Total DNA maxiprep was obtained using KingFisher Cell and tissue DNA kit (Thermo Fisher Scientific, France) according to the manufacturer’s instructions. DNA quantitation was done using the ND-1000 spectrophotometer (NanoDrop Technologies Inc., Wilmington, USA), and DNA quality was controlled using the Qubit 3.0 Fluorometer (Invitrogen, France). Sequencing was carried out by the Mutualized Platform for Microbiology (P2M) at Institut Pasteur (France). DNA whole-genome shotgun sequencing libraries were prepared using the Nextera XT kit (Illumina), and 2×150 bp paired-end sequencing was performed using an Illumina NextSeq500 instrument, yielding 5 374 051 and 2 684 746 read pairs for strains cfHf56-1^T^ (307× average sequencing depth and 349 bp average insert size) and SW 252 (154× and 372 bps), respectively. Read processing, genome assembly and gene annotation were performed using fq2dna version 21.06 (https://gitlab.pasteur.fr/GIPhy/fq2dna). The draft genome of strain cfHf56-1^T^ had 5 176 336 bps on 105 contigs (NG50, 172 465, G+C content, 40.48%). The draft genome of strain SW 252 had 5 192 604 bps on 117 contigs (NG50, 169 085, G+C content, 40.51%). A total of 4082 and 4114 coding sequences were inferred from strains cfHf56-1^T^ and SW 252, respectively. Genome sequence authentication was assessed by aligning the 16S rRNA segment derived from Sanger sequencing (MN942006) against both *de novo* assemblies (CAMAPC00000000 and CAMAPD00000000, respectively) using blastn, leading to >99.8 % pairwise sequence identities. The completeness/contamination indices were assessed as 100.00 %/0.96% and 99.72 %/1.01 % for the draft genomes of cfHf56-1^T^ and SW 252, respectively, using CheckM version 1.1.3 [23].

**Fig. 2.**
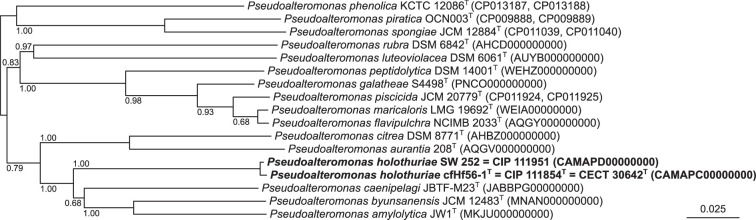
Phylogenetic subtree showing the phylogenetic placement of strains cfHf56-1^T^ and SW 252 among 15 closely related *Pseudoalteromonas* type strains. The complete ME whole-genome-based tree was inferred using JolyTree (Fig. S1). Genome sequence accessions are indicated between parentheses. Branch supports (>0.5) were assessed by the rate of elementary quartets, as estimated by JolyTree. Bar, 0.025 nucleotide substitutions per site.

Pairwise average nucleotide and amino acid identity (ANI and AAI, respectively) values were computed using OGRI_B version 1.2 (https://gitlab.pasteur.fr/GIPhy/OGRI) between the draft genomes of both strains cfHf56-1^T^ and SW 252 and 46 publicly available *Pseudoalteromonas* type strain genomes (Table S1, available in the online version of this article). We used the genome of *Pseudoalteromonas haloplanktis* CIP 103197^T^ as that of the type species. ANI and AAI values are reported in Table 1 for six selected genomes, along with the associated digital DNA–DNA hybridization (dDDH) values (Formula 2; https://ggdc.dsmz.de). All the estimated pairwise similarity values are close to 100% between the draft genomes of cfHf56-1^T^ and SW 252, whereas they are far below the commonly admitted delineation cutoffs (e.g. ANI, 95%; AAI, 95%; and dDDH, 70%) against every other *Pseudoalteromonas* type strain genomes [24,26]. Finally, a phylogenomic classification of the *Pseudoalteromonas* genome sequences was also inferred using JolyTree version 2.0 [27,28], such a tree confirming that both strains cfHf56-1^T^ and SW 252 formed a stable cluster (100% branch support) with the type strains of *P. caenipelagi*, *P. amylolytica* and *P. byunsanensis* (Figs 2 and S1).

**Table 1.** Pairwise ANI (lower triangular) and dDDH (upper triangular) values between selected genomes

	cfHf56-1^T^(= CIP 111854^T^)	SW 252(= CIP 111951)	*P. byunsanensis*JCM 12483^T^	*P. caenipelagi*JBTF-M23^T^	*P. amylolytica*JW1^T^	*P. haloplanktis*CIP 103197^T^
**cfHf56-1^T^ (= CIP 111854^T^)**		96.00	19.80	20.80	19.70	19.60
SW 252	99.50		19.90	20.80	19.80	19.60
JCM 12483^T^	75.90	75.99		19.90	26.40	20.30
JBTF-M23^T^	77.47	77.40	76.22		19.90	19.60
JW1^T^	75.73	75.79	83.51	76.04		20.10
CIP 103197^T^	71.76	71.98	71.42	71.64	71.44	

## Physiology and chemotaxonomy

All phenotypic tests were performed simultaneously with cfHf56-1^T^, SW 252 and the closely related type strains *P. caenipelagi* CIP 111909^T^, *P. byunsanensis* CIP 109024^T^ and *P. amylolytica* CIP 111901^T^. Gram-staining was done by using a kit and a centrifuge system (Previ Color Gram, bioMérieux, Marcy l’Etoile, France) according to the manufacturer’s instructions. Cell morphology and motility were examined by light microscopy. The shape and color of the colonies were observed on MA plates after a 48-h incubation period at 25 °C. Growth was observed at various temperatures (4 °C, 18 °C, 25 °C, 30 °C, 37 °C and 45 °C) in MB with gentle shaking at 100 r.p.m. for up to 10 days. The pH range (5–10) for growth (at intervals of 0.5 pH units) was carried out by adding HCl (1 N) or NaOH (1 N) to buffered MB at 25 °C, using citric acid/sodium citrate buffer, di-potassium hydrogen phosphate/potassium di-hydrogen phosphate and sodium carbonate/di-sodium carbonate as buffering systems. A medium that contained (per liter) 5 g MgCl_2_, 2 g MgSO_4_, 0.5 g CaCl_2_, 1 g KCl, 5 g peptone and various amounts of NaCl (0, 0.5, 1, 2, 3, 4, 6, 8, 10, 15 and 20 % w/v) was used to determine NaCl requirements and tolerance. Antibiotic susceptibility testing was done according to the Kirby–Bauer disc diffusion method on MA plates after 48 h incubation at 25 °C with the following commercial compounds (µg per disc): trimethoprim-sulphamethoxazole (25), flumequin (30), enrofloxacin (5), erythromycin (15), streptomycin (10), gentamicin (30), chloramphenicol (30), neomycin (30), cephalothin (30), lincomycin (15), nitrofurantoin (10), tetracycline (30) and novobiocin (5). Catalase and oxidase activities were determined by bubble production in the presence of H_2_O_2_ and using a commercial strip (Merck Millipore), respectively. Biochemical properties such as substrate assimilation or enzyme productions were determined with the API 20NE and API ZYM galleries (bioMérieux) according to the manufacturer’s instructions, except for the resuspension medium which was supplemented by 2% (w/v) sea salts [3]. Both galleries were incubated for 48 h at optimal growth temperature, either at 25 °C for strain SW 252 or 30 °C for the four other strains. Similarly, fatty acid methyl esters (FAMEs) were prepared from the biomass of the five tested strains grown on MA at their optimal temperature [29]. Analysis of FAME extracts was performed by gas chromatography (GC model 7890B, Agilent, USA) with an FID detector [30]. The identity of fatty acids was verified by a gas chromatography–mass spectrometry system (model 8890 GC/5977B MSD, Agilent).

**Fig. 3.**
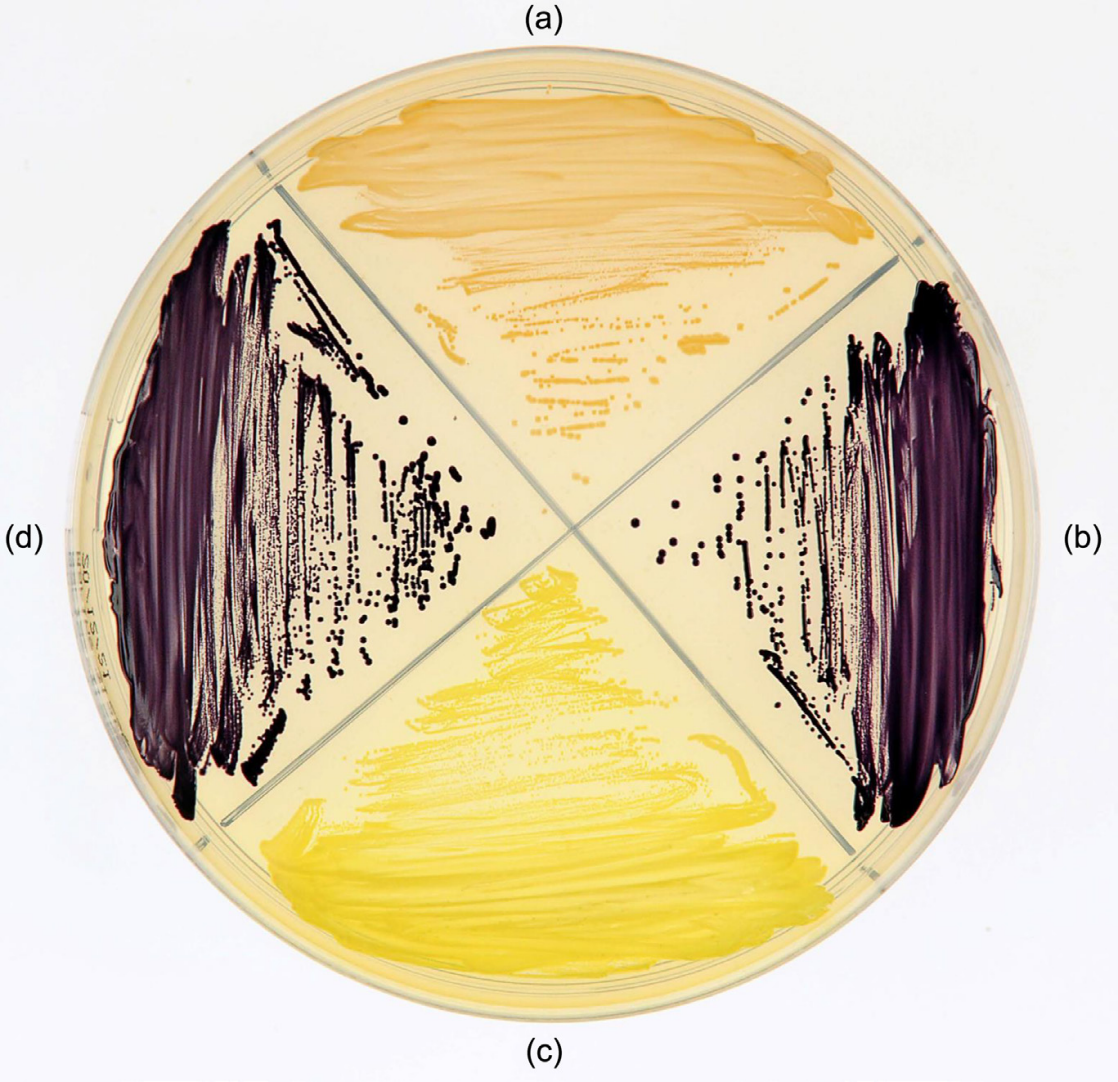
Pictures of *P. holothuriae* cfHf56-1^T^ and pigmented related strains on MA plate after 2 days of incubation at 25 °C. (a): cfHf56-1^T^ (= CIP 11854^T^); (b): *P. byunsanensis* CIP 109024^T^; (c): *P. caenipelagi* CIP 111909^T^ and (d): *P. amylolytica* CIP 111901^T^.

Strains cfHf56-1^T^ and SW 252 are Gram-stain-negative, aerobic, motile and non-spore-forming rods. They were observed to be not able to grow at 4 °C and 37 °C. The pH range for optimal growth was 7–8 at 25 °C. Type strain cfHF56-1^T^ was found to grow optimally in the presence of 2% NaCl at 30 °C. Colonies appeared in weak brown color (Fig. 3), round, convex, opaque and regular, sizing between 3 and 4 mm in diameter. The distinctive characteristics of the two studied strains with related *Pseudoalteromonas* type strains have been reported in Table 2, but many phenotypic traits were common. All tested strains were observed as positive for catalase and oxidase activities, utilization of *N*-acetyl-glucosamine and d-maltose, gelatin hydrolysis, presence of both acid and alkaline phosphatase enzymes, leucine arylamidase and naphthol-AS-BI-phosphohydrolase activities. There was a delayed utilization of d-mannitol. All strains were observed as negative for acid production from glucose, nitrate reduction, utilization of l-tryptophan, l-arginine, urea, potassium gluconate, capric acid, adipic acid, malic acid, trisodium citrate or phenylacetic acid. A clear zone of growth inhibition, at least greater than 5 mm around the disc, was observed repeatedly for erythromycin, chloramphenicol, flumequin, enrofloxacin, streptomycin, gentamicin, neomycin and trimethoprim-sulfamethoxazole antibiotic discs.

**Table 2.** Distinctive characteristics of strains cfHf56-1^T^ (= CIP 111854^T^), SW 252 (= CIP 111951) and the type strains of the three closely related *Pseudoalteromonas* species; 1, cfHf56-1^T^ (= CIP 111854^T^); 2, SW 252 (= CIP 111951); 3, *P. caenipelagi* CIP 111909^T^ ; 4, *P. byunsanensis* CIP 109024^T^ ; 5, *P. amylolytica* CIP 111901^T^. Data obtained from this study. + : positive; (+) : weakly positive; – : negative

Characteristics	1	2	3	4	5
DNA G+C content (mol%)	40.5	40.5	42.0	42.4	43.3
Pigmentation	+Brown	+Brown	+Yellow	+Purple	+Purple
Growth at 37 °C	−	−	+	+	+
**Growth with**					
0.5 % (w/v) NaCl	−	−	+	+	+
8.0 % (w/v) NaCl	+	+	−	−	+
pH range for growth	6–9	6–9	5–7.5	5–10	6–10.5
**Hydrolysis of**					
Aesculin	(+)	(+)	+	+	+
Gelatin	(+)	+	+	+	+
**Enzymatic activity**					
Valine arylamidase	+	+	−	−	−
Esterase (C4)	−	(+)	−	−	−
Esterase lipase (C8)	+	+	(+)	(+)	+
Trypsin	+	+	+	+	−
*N*-acetyl-β-glucosaminidase	+	+	+	−	−
Lipase (C14)	−	−	+	−	+
**Utilization of**					
d-Glucose	+	+	+	+	(+)
l-Arabinose	+	+	+	+	(+)
d-Mannose	(+)	(+)	+	(+)	(+)

On the basis of these results, the brown pigment, the absence of growth at 37 °C and the detection of valine arylamidase activity were three clear-cut phenotypic traits that could distinguish both cfHf56-1^T^ and SW252 from the three other type strains, * P. caenipelagi* CIP 111909^T^, *P. byunsanensis* CIP 109024^T^ and *P. amylolytica* CIP 111901^T^. In contrast, all five strains exhibited similar fatty acid profiles (Table 3). The predominant lipids (>5 %) detected in strain cfHf56-1^T^ were 16 : 1 cis9 (30.6%), 16 : 0 (20.9%), 16 : 1 cis10 (7.3%), 18 : 1 cis11 (6.0%) and 17 : 1 cis9 (5.9%). This fatty acid profile is in accordance with the ones previously reported for *P. caenipelagi*, *P. byunsanesis* and *P. amylolytica* [3,21].

**Table 3.** Cellular fatty acid composition (%) of strains cfHf56-1^T^ (= CIP 111854^T^), SW 252 (= CIP 111951) and the type strains of three closely related *Pseudoalteromonas* species; 1, cfHf56-1^T^ (= CIP 111854^T^); 2, SW 252 (= CIP 111951); 3, *P. caenipelagi* CIP 111909^T^; 4, *P. byunsanensis* CIP 109024^T^; 5 *P*. *amylolytica* CIP 111901^T^. All data obtained from this study. Fatty acids that represented <1.0% for all strains are not given. –: Not detected

Characteristics	1	2	3	4	5
**Straight chain**					
12 : 0	1.5	1.8	2.2	0.9	0.7
14 : 0	1.2	0.9	3.2	2.4	1.9
15 : 0	3.2	3.2	2.3	2.2	3.2
16 : 0	20.9	13.3	8.9	17.9	12.2
17 : 0	5.0	3.4	0.9	1.7	2.1
18 : 0	1.0	0.4	0.3	0.6	–
**Unsaturated**					
14 : 1 cis7	0.5	0.8	1.1	0.4	1.0
15 : 1 cis6	2.0	3.2	0.9	1.5	1.8
16 : 1 cis9	30.6	27.7	42.5	33.0	19.8
16 : 1 cis10	7.3	10.2	6.8	12.5	29.6
17 : 1 cis9	5.9	9.5	1.5	5.7	4.5
18 : 1 cis11	6.9	6.4	2.6	6.2	6.2
18 : 1 cis12	1.4	–	0.5	0.6	2.7
**Hydroxy**					
10 : 0 3-OH	1.7	3.3	6.6	1.9	2.8
11 : 0 3-OH	1.0	3.7	2.7	1.0	1.5
12 : 0 3-OH	3.6	4.7	8.7	3.1	1.7
14 : 0 2-OH	1.3	–	–	1.4	–
**Branched**					
16 : 0 iso	–	–	–	1.0	–
ECL 11.762*	1.8	3.0	6.2	2.5	2.5

*Unknown compound with equivalent chain length 11.762.

## Taxonomic conclusions

To sum up, the combination of genotypic, phenotypic and chemotaxonomic results reported here for the two strains cfHf56-1^T^ and SW 252 gives credit to assign them as members of the genus *Pseudoalteromonas*. They are distinguished from the type strains of *P. caenipelagi*, *P. byunsanensis* and *P. amylolytica* by three phenotypic characters: the brown pigment, the absence of growth at 37 °C and the detection of valine arylamidase activity. Their genetic distinctiveness by the estimated ANI, AAI and dDDH values support unambiguously that strains cfHf56-1^T^ (= CIP 111854^T^ = CECT 30642^T^) and SW 252 (= CIP 111951) represent a novel species within the genus *Pseudoalteromonas,* for which the name *Pseudoalteromonas holothuriae* sp. nov. is proposed.

## Description of *Pseudoalteromonas holothuriae* sp. nov.

*Pseudoalteromonas holothuriae* (ho.lo.thu'ri.ae. N.L. gen. n. *holothuriae*, of the sea cucumber genus *Holothuria*).

The cells are Gram-stain-negative, non-spore-forming, motile and rod-shaped (1–2 µm in length). After 48 hours incubation at 25 °C on MA, bacterial cells form circular weak brown colonies with translucent margin, sizing between 3 and 4 mm in diameter. Optimal growth temperature is 25–30 °C. Growth occurs at 18 °C but not at 4 °C or 37 °C. The optimal pH for growth is 7–8. The strain tolerates up to 8% NaCl (w/v) and requires at least 1% with an optimum of 2 % at 30 °C for the type strain. Tests for catalase and oxidase activities are positive. Tests are also positive for alkaline phosphatase, esterase lipase (C8), valine arylamidase, leucine arylamidase, acid phosphatase, trypsin, *N*-acetyl-*β*-glucosaminidase and naphthol-AS-BI-phosphohydrolase. Negative for urease, *α*-glucosidase, *β*-glucosidase, *α*-galactosidase, *β*-galactosidase, *β*-glucuronidase, cysteine arylamidase, esterase (C4), lipase (C14), *α*-mannosidase, *α*-fucosidase, *α*-chymotrypsin and arginine dihydrolase. No reduction of nitrates to nitrites detected. Not able to ferment glucose. Aesculin and gelatin can be hydrolyzed. d-Glucose, l-arabinose, d-mannose, d-maltose, d-mannitol and *N*-acetyl-glucosamine can be assimilated, but not potassium gluconate, capric acid, adipic acid, malic acid, trisodium citrate and phenylacetic acid. Major fatty acids (>5 %) are 16 : 1 cis9, 16 : 0, 16 : 1 cis10, 18 : 1 cis11 and 17 : 1 cis9.

The type strain, cfHf56-1^T^ (= CIP 111854^T^ = CECT 30642^T^), was isolated from the coelomic fluid of the sea cucumber *Holothuria forskali*. The GenBank/EMBL/DDBJ accession number for the 16S rRNA gene sequence is MN942006. The draft genome is characterized by a size of 5.1 Mbp and a G+C content of 40.5%. The GenBank/EMBL/DDBJ accession number of the genome assembly is CAMAPC00000000.

## Supplementary material

10.1099/ijsem.0.006601 Uncited Supplementary Material 1.

## Abbreviations

AAI: average amino-acid identity
ANI: average nuclotide identity
dDDH: digital DNA–DNA hybridization
FAME: fatty acid methyl esters
MB: marine broth
ML: maximum likelihood
MP: maximum parsimony
