# Supplementary material for: Pseudoalteromonas holothuriae sp. nov., isolated from the sea cucumber Holothuria forskali
Source: Int J Syst Evol Microbiol. 2025 Feb 24;75(2):006601. doi: 10.1099/ijsem.0.006601 (PMC12281933; doi:10.1099/ijsem.0.006601)
Supplement: Uncited Supplementary Material 1. [file ijsem-75-06601-s001.pdf]

Supplementary material

Figure S1

Table S1

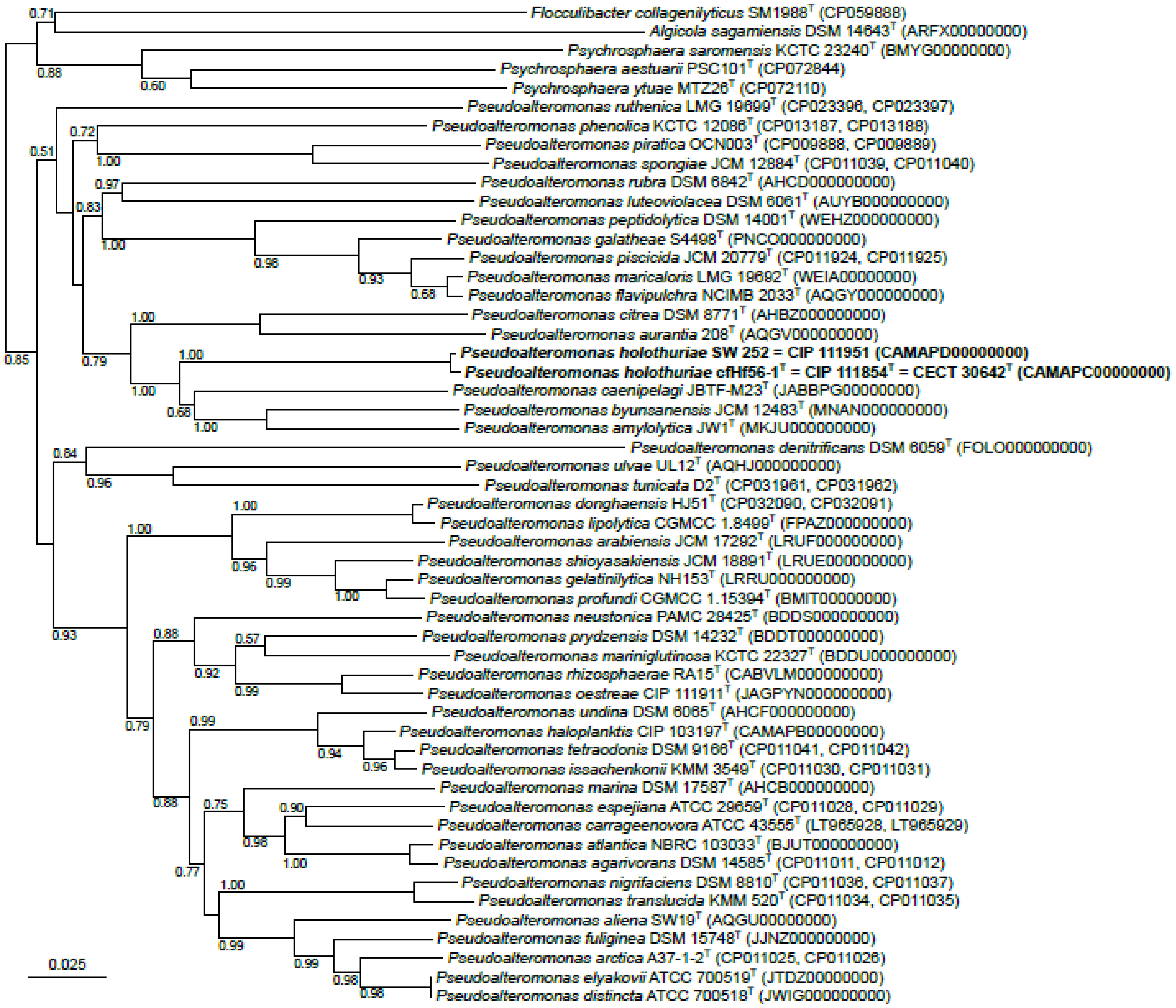

Figure S1. Whole-genome-based phylogenetic tree showing the phylogenetic placement of strains cfHf56-1T and SW 252 among 46 *Pseudoalteromonas* type strains. This minimum evolution (ME) tree was inferred using JolyTree. Five *Algicola*, *Flocculibacter* and *Psychrosphaera* type strain genomes were used as an outgroup. Genome sequence accessions are indicated between parentheses. Branch supports (>0.5) were assessed by the rate of elementary quartets, as estimated by JolyTree. Bar, 0.025 nucleotide substitutions per site

Table S1. Listing of ANI, AAI, and dDDH values between the draft genomes of both strains **cfHf56-1^T (A)** and **SW 252 (B),** and 46 publicly available *Pseudoalteromonas* type strain genomes.

(A) Strain *cfHf56-1(T) = CIP111854(T)*

	ACCESSION	ANI [CI]	AAI [CI]	dDDH [CI]
Pseudoalteromonas holothuriae cfHf56-1(T) = CIP111854(T)	CAMAPC00000000	99.50 [99.44-99.56]	99.49 [99.36-99.57]	96.00 [94.7-97.1]
Pseudoalteromonas caenipelagi JBTF-M23(T)	JABBPG00000000	77.40 [77.20-77.61]	83.68 [83.29-84.05]	20.80 [18.6-23.3]
Pseudoalteromonas byunsanensis JCM12483(T)	MNAN000000000	75.99 [75.72-76.18]	81.98 [81.57-82.42]	19.90 [17.7-22.3]
Pseudoalteromonas amylolytica JW1(T)	MKJU000000000	75.79 [75.56-76.01]	81.95 [81.48-82.40]	19.80 [17.6-22.2]
Pseudoalteromonas citrea DSM8771(T)	AHBZ000000000	73.14 [72.87-73.41]	75.27 [74.76-75.83]	19.60 [17.4-22.0]
Pseudoalteromonas aurantia 208(T)	AQGV000000000	72.79 [72.54-73.05]	74.81 [74.24-75.29]	19.70 [17.5-22.1]
Pseudoalteromonas phenolica KCTC12086(T)	CP013187 CP013188	72.23 [71.88-72.52]	73.50 [72.91-73.99]	19.90 [17.7-22.3]
Pseudoalteromonas haloplanktis CIP103197(T)	CAMAPB00000000	71.98 [71.68-72.30]	71.61 [71.12-72.16]	19.60 [17.4-22.0]
Pseudoalteromonas gelatinilytica NH153(T)	LRRU000000000	71.90 [71.66-72.14]	71.70 [71.19-72.30]	19.60 [17.4-22.0]
Pseudoalteromonas distincta ATCC700518(T)	JWIG000000000	71.85 [71.54-72.14]	71.32 [70.70-71.86]	20.60 [18.4-23.0]
Pseudoalteromonas issachenkonii KMM3549(T)	CP011030 CP011031	71.83 [71.54-72.12]	71.43 [70.91-71.98]	20.60 [18.4-23.0]
Pseudoalteromonas oestreae CIP111911(T)	JAGPYN000000000	71.82 [71.51-72.12]	71.36 [70.86-71.92]	20.50 [18.2-22.9]
Pseudoalteromonas shioyasakiensis JCM18891(T)	LRUE000000000	71.81 [71.48-72.09]	71.71 [71.15-72.31]	19.70 [17.5-22.1]
Pseudoalteromonas nigrifaciens DSM8810(T)	CP011036 CP011037	71.81 [71.48-72.08]	71.61 [71.16-72.16]	20.50 [18.3-23.0]
Pseudoalteromonas tetraodonis DSM9166(T)	CP011041 CP011042	71.81 [71.47-72.08]	71.55 [70.99-72.13]	20.30 [18.1-22.7]
Pseudoalteromonas arabiensis JCM17292(T)	LRUF000000000	71.75 [71.45-72.02]	71.83 [71.31-72.40]	20.10 [17.9-22.6]
Pseudoalteromonas arctica A37-1-2(T)	CP011025 CP011026	71.73 [71.47-72.03]	71.35 [70.78-71.88]	20.50 [18.3-22.9]
Pseudoalteromonas profundi CGMCC 1.15394(T)	BMIT00000000	71.70 [71.36-71.97]	71.71 [71.14-72.26]	19.70 [17.5-22.1]
Pseudoalteromonas carrageenovora ATCC43555(T)	LT965928 LT965929	71.70 [71.39-71.97]	71.31 [70.83-71.95]	20.30 [18.1-22.7]
Pseudoalteromonas espejiana ATCC29659(T)	CP011028 CP011029	71.70 [71.38-72.01]	71.45 [70.91-72.11]	20.10 [17.9-22.6]
Pseudoalteromonas mariniglutinosa KCTC22327(T)	BDDU000000000	71.68 [71.36-71.92]	71.57 [71.08-72.06]	19.50 [17.3-21.9]
Pseudoalteromonas lipolytica CGMCC1.8499(T)	FPAZ000000000	71.67 [71.38-71.93]	71.78 [71.16-72.32]	19.80 [17.6-22.2]
Pseudoalteromonas elyakovii ATCC700519(T)	JTDZ00000000	71.67 [71.40-71.95]	71.30 [70.71-71.89]	20.30 [18.1-22.7]
Pseudoalteromonas translucida KMM520(T)	CP011034 CP011035	71.66 [71.33-71.95]	71.60 [71.03-72.10]	20.50 [18.3-22.9]
Pseudoalteromonas agarivorans DSM14585(T)	CP011011 CP011012	71.66 [71.36-71.94]	71.49 [70.97-72.01]	20.50 [18.3-23.0]
Pseudoalteromonas fuliginea DSM15748(T)	JJNZ000000000	71.65 [71.39-71.93]	70.90 [70.29-71.47]	19.70 [17.5-22.1]
Pseudoalteromonas donghaensis HJ51(T)	CP032090 CP032091	71.65 [71.40-71.93]	71.81 [71.21-72.37]	20.60 [18.3-23.0]
Pseudoalteromonas atlantica NBRC103033(T)	BJUT000000000	71.65 [71.38-71.93]	71.53 [70.82-72.02]	19.40 [17.2-21.8]
Pseudoalteromonas undina DSM6065(T)	AHCF000000000	71.64 [71.35-71.94]	71.21 [70.70-71.70]	20.40 [18.2-22.9]
Pseudoalteromonas piscicida JCM20779(T)	CP011924 CP011925	71.58 [71.33-71.84]	72.10 [71.52-72.75]	20.30 [18.1-22.7]
Pseudoalteromonas prydzensis DSM14232(T)	BDDT000000000	71.56 [71.31-71.86]	71.43 [70.76-71.94]	19.30 [17.1-21.6]
Pseudoalteromonas maricaloris LMG19692(T)	WEIA00000000	71.54 [71.29-71.86]	72.11 [71.55-72.63]	19.70 [17.5-22.1]
Pseudoalteromonas neustonica PAMC28425(T)	BDDS000000000	71.54 [71.22-71.83]	70.91 [70.32-71.46]	19.20 [17.0-21.6]
Pseudoalteromonas marina DSM17587(T)	AHCB000000000	71.46 [71.20-71.70]	71.21 [70.61-71.91]	19.40 [17.2-21.8]
Pseudoalteromonas galatheae S4498(T)	PNCO000000000	71.46 [71.18-71.75]	72.28 [71.72-72.73]	19.80 [17.6-22.2]
Pseudoalteromonas rhizosphaerae RA15(T)	CABVLM000000000	71.46 [71.16-71.71]	71.10 [70.54-71.61]	19.60 [17.4-22.0]
Pseudoalteromonas flavipulchra NCIMB2033(T)	AQGY000000000	71.39 [71.13-71.65]	72.07 [71.46-72.61]	19.50 [17.3-21.9]
Pseudoalteromonas aliena SW19(T)	AQGU00000000	71.31 [71.05-71.64]	70.70 [70.10-71.22]	19.80 [17.6-22.2]
Pseudoalteromonas peptidolytica DSM14001(T)	WEHZ000000000	71.30 [71.08-71.56]	72.33 [71.73-72.81]	19.60 [17.4-22.0]
Pseudoalteromonas luteoviolacea DSM6061(T)	AUYB000000000	70.88 [70.59-71.16]	71.47 [70.77-72.05]	19.10 [17.0-21.5]
Pseudoalteromonas rubra DSM6842(T)	AHCD000000000	70.64 [70.40-70.91]	71.85 [71.39-72.38]	19.30 [17.1-21.7]
Pseudoalteromonas tunicata D2(T)	CP031961 CP031962	70.23 [69.93-70.50]	67.87 [67.32-68.54]	20.70 [18.5-23.1]
Pseudoalteromonas piratica OCN003(T)	CP009888 CP009889	70.05 [69.75-70.32]	67.16 [66.43-67.73]	22.40 [20.1-24.8]
Pseudoalteromonas spongiae JCM12884(T)	CP011039 CP011040	69.95 [69.56-70.24]	67.18 [66.59-67.69]	23.00 [20.7-25.5]
Pseudoalteromonas ulvae UL12(T)	AQHJ000000000	69.91 [69.69-70.20]	68.10 [67.55-68.62]	20.50 [18.3-22.9]
Pseudoalteromonas ruthenica LMG19699(T)	CP023396 CP023397	69.84 [69.54-70.20]	69.41 [68.84-69.96]	21.20 [19.0-23.6]
Pseudoalteromonas denitrificans DSM6059(T)	FOLO000000000	69.67 [69.40-69.95]	66.44 [65.83-66.99]	18.80 [16.6-21.2]

(B), Strain *SW 252 = CIP111951*

GENOME	ACCESSION	ANI [CI]	AAI [CI]	dDDH [CI]
Pseudoalteromonas holothuriae SW 252 = CIP111951	CAMAPD00000000	99.50 [99.44-99.55]	99.49 [99.37-99.59]	96.00 [94.7-97.1]
Pseudoalteromonas caenipelagi JBTF-M23(T)	JABBPG00000000	77.47 [77.25-77.67]	83.66 [83.25-84.01]	20.80 [18.6-23.2]
Pseudoalteromonas byunsanensis JCM12483(T)	MNAN000000000	75.90 [75.70-76.14]	81.99 [81.55-82.45]	19.80 [17.6-22.2]
Pseudoalteromonas amylolytica JW1(T)	MKJU000000000	75.73 [75.49-75.98]	81.90 [81.50-82.31]	19.70 [17.5-22.1]
Pseudoalteromonas citrea DSM8771(T)	AHBZ000000000	73.22 [72.94-73.48]	75.18 [74.60-75.61]	19.50 [17.3-21.9]
Pseudoalteromonas aurantia 208(T)	AQGV000000000	72.75 [72.47-73.05]	74.81 [74.15-75.33]	19.60 [17.4-22.0]
Pseudoalteromonas phenolica KCTC12086(T)	CP013187 CP013188	72.33 [72.04-72.57]	73.50 [72.99-74.12]	19.70 [17.5-22.1]
Pseudoalteromonas nigrifaciens DSM8810(T)	CP011036 CP011037	71.91 [71.66-72.20]	71.56 [70.92-72.13]	20.60 [18.3-23.0]
Pseudoalteromonas gelatinilytica NH153(T)	LRRU000000000	71.87 [71.63-72.20]	71.71 [71.16-72.22]	19.70 [17.5-22.1]
Pseudoalteromonas tetraodonis DSM9166(T)	CP011041 CP011042	71.80 [71.47-72.08]	71.55 [70.90-72.11]	20.40 [18.2-22.9]
Pseudoalteromonas distincta ATCC700518(T)	JWIG000000000	71.79 [71.47-72.07]	71.37 [70.77-71.97]	20.90 [18.6-23.3]
Pseudoalteromonas carrageenovora ATCC43555(T)	LT965928 LT965929	71.77 [71.44-72.04]	71.28 [70.67-71.76]	20.50 [18.2-22.9]
Pseudoalteromonas haloplanktis CIP103197(T)	CAMAPB00000000	71.76 [71.52-72.06]	71.69 [71.17-72.36]	19.60 [17.4-22.0]
Pseudoalteromonas arctica A37-1-2(T)	CP011025 CP011026	71.74 [71.48-72.01]	71.30 [70.59-71.82]	20.60 [18.4-23.0]
Pseudoalteromonas arabiensis JCM17292(T)	LRUF000000000	71.74 [71.44-72.02]	71.82 [71.24-72.33]	20.10 [17.9-22.5]
Pseudoalteromonas mariniglutinosa KCTC22327(T)	BDDU000000000	71.73 [71.43-71.99]	71.57 [71.03-72.14]	19.50 [17.3-21.9]
Pseudoalteromonas agarivorans DSM14585(T)	CP011011 CP011012	71.73 [71.45-72.04]	71.53 [70.93-72.12]	20.60 [18.3-23,3]
Pseudoalteromonas oestreae CIP111911(T)	JAGPYN000000000	71.72 [71.41-71.98]	71.41 [70.85-71.95]	20.40 [18.2-22.8]
Pseudoalteromonas translucida KMM520(T)	CP011034 CP011035	71.72 [71.41-71.97]	71.57 [71.06-72.18]	20.50 [18.2-22.9]
Pseudoalteromonas shioyasakiensis JCM18891(T)	LRUE000000000	71.69 [71.38-71.93]	71.71 [71.18-72.30]	19.80 [17.6-22.2]
Pseudoalteromonas issachenkonii KMM3549(T)	CP011030 CP011031	71.68 [71.42-71.94]	71.37 [70.69-72.02]	20.60 [18.4-23.0]
Pseudoalteromonas fuliginea DSM15748(T)	JJNZ000000000	71.68 [71.37-72.03]	70.94 [70.29-71.41]	19.70 [17.5-22.1]
Pseudoalteromonas donghaensis HJ51(T)	CP032090 CP032091	71.66 [71.32-71.95]	71.83 [71.27-72.29]	20.50 [18.3-22.9]
Pseudoalteromonas profundi CGMCC 1.15394(T)	BMIT00000000	71.65 [71.36-71.91]	71.69 [71.09-72.20]	19.50 [17.3-21.9]
Pseudoalteromonas espejiana ATCC29659(T)	CP011028 CP011029	71.64 [71.37-71.90]	71.45 [70.92-71.99]	20.20 [18.0-22.6]
Pseudoalteromonas undina DSM6065(T)	AHCF000000000	71.64 [71.37-71.89]	71.15 [70.54-71.67]	20.60 [18.3-23.0]
Pseudoalteromonas lipolytica CGMCC1.8499(T)	FPAZ000000000	71.63 [71.32-71.94]	71.85 [71.21-72.50]	19.90 [17.6-22.3]
Pseudoalteromonas elyakovii ATCC700519(T)	JTDZ00000000	71.62 [71.31-71.91]	71.36 [70.87-71.94]	20.40 [18.2-22.8]
Pseudoalteromonas atlantica NBRC103033(T)	BJUT000000000	71.56 [71.30-71.87]	71.56 [70.96-72.07]	19.40 [17.2-21.8]
Pseudoalteromonas galatheae S4498(T)	PNCO000000000	71.48 [71.22-71.74]	72.34 [71.81-72.86]	20.10 [17.9-22.5]
Pseudoalteromonas neustonica PAMC28425(T)	BDDS000000000	71.48 [71.21-71.74]	70.97 [70.34-71.44]	19.30 [17.1-21.6]
Pseudoalteromonas marina DSM17587(T)	AHCB000000000	71.48 [71.17-71.79]	71.19 [70.64-71.78]	19.50 [17.3-21.9]
Pseudoalteromonas rhizosphaerae RA15(T)	CABVLM000000000	71.47 [71.17-71.73]	71.10 [70.53-71.65]	19.50 [17.3-21.9]
Pseudoalteromonas aliena SW19(T)	AQGU00000000	71.45 [71.12-71.70]	70.76 [70.17-71.31]	19.70 [17.5-22.1]
Pseudoalteromonas piscicida JCM20779(T)	CP011924 CP011925	71.41 [71.12-71.67]	72.07 [71.49-72.59]	20.30 [18.1-22.7]
Pseudoalteromonas prydzensis DSM14232(T)	BDDT000000000	71.40 [71.11-71.68]	71.39 [70.85-71.98]	19.20 [17.0-21.6]
Pseudoalteromonas maricaloris LMG19692(T)	WEIA00000000	71.33 [71.07-71.60]	72.06 [71.56-72.65]	19.70 [17.5-22.1]
Pseudoalteromonas flavipulchra NCIMB2033(T)	AQGY000000000	71.28 [71.01-71.53]	72.01 [71.54-72.51]	19.50 [17.3-21.9]
Pseudoalteromonas peptidolytica DSM14001(T)	WEHZ000000000	71.23 [70.94-71.46]	72.33 [71.77-72.85]	19.70 [17.5-22.1]
Pseudoalteromonas luteoviolacea DSM6061(T)	AUYB000000000	70.87 [70.59-71.12]	71.45 [70.97-71.99]	19.20 [17.0-21.6]
Pseudoalteromonas rubra DSM6842(T)	AHCD000000000	70.43 [70.18-70.67]	71.87 [71.21-72.33]	19.30 [17.1-21.7]
Pseudoalteromonas tunicata D2(T)	CP031961 CP031962	70.20 [69.92-70.49]	67.89 [67.34-68.48]	20.80 [18.5-23.2]
Pseudoalteromonas spongiae JCM12884(T)	CP011039 CP011040	70.10 [69.80-70.36]	67.30 [66.65-67.82]	23.50 [21.2-25.9]
Pseudoalteromonas piratica OCN003(T)	CP009888 CP009889	70.03 [69.74-70.37]	67.16 [66.60-67.82]	22.40 [20.2-24.9]
Pseudoalteromonas ulvae UL12(T)	AQHJ000000000	70.01 [69.70-70.30]	68.12 [67.39-68.69]	20.40 [18.2-22.8]
Pseudoalteromonas denitrificans DSM6059(T)	FOLO000000000	69.97 [69.67-70.21]	66.54 [65.98-67.19]	18.90 [16.8-21.3]
Pseudoalteromonas ruthenica LMG19699(T)	CP023396 CP023397	69.90 [69.66-70.17]	69.42 [68.76-69.99]	21.00 [18.7-23.4]
